# Fear Conditioning Induced by Interpersonal Conflicts in Healthy Individuals

**DOI:** 10.1371/journal.pone.0125729

**Published:** 2015-05-15

**Authors:** Mitsuhiro Tada, Hiroyuki Uchida, Takaki Maeda, Mika Konishi, Satoshi Umeda, Yuri Terasawa, Shinichiro Nakajima, Masaru Mimura, Tomoyuki Miyazaki, Takuya Takahashi

**Affiliations:** 1 Department of Neuropsychiatry, Keio University School of Medicine, Tokyo, Japan; 2 Geriatric Psychiatry Division, Centre for Addiction and Mental Health, Toronto, Ontario, Canada; 3 Department of Psychology, Keio University, Tokyo, Japan; 4 Centre for Advanced Research on Logic and Sensibility, Keio University, Tokyo, Japan; 5 Department of Psychophysiology, National Center of Neurology and Psychiatry, Tokyo, Japan; 6 Multimodal Imaging Group—Research Imaging Centre, Centre for Addiction and Mental Health, Toronto, Ontario, Canada; 7 Department of Psychiatry, University of Toronto, Toronto, Ontario, Canada; 8 Department of Physiology, Yokohama City University Graduate School of Medicine, Yokohama, Japan; 9 Dominick P. Purpura Department of Neuroscience, Albert Einstein College of Medicine, New York, New York, United States of America; Tokai University, JAPAN

## Abstract

Psychophysiological markers have been focused to investigate the psychopathology of psychiatric disorders and personality subtypes. In order to understand neurobiological mechanisms underlying these conditions, fear-conditioning model has been widely used. However, simple aversive stimuli are too simplistic to understand mechanisms because most patients with psychiatric disorders are affected by social stressors. The objective of this study was to test the feasibility of a newly-designed conditioning experiment using a stimulus to cause interpersonal conflicts and examine associations between personality traits and response to that stimulus. Twenty-nine healthy individuals underwent the fear conditioning and extinction experiments in response to three types of stimuli: a simple aversive sound, disgusting pictures, and pictures of an actors’ face with unpleasant verbal messages that were designed to cause interpersonal conflicts. Conditioned response was quantified by the skin conductance response (SCR). Correlations between the SCR changes, and personality traits measured by the Zanarini Rating Scale for Borderline Personality Disorder (ZAN-BPD) and Revised NEO Personality Inventory were explored. The interpersonal conflict stimulus resulted in successful conditioning, which was subsequently extinguished, in a similar manner as the other two stimuli. Moreover, a greater degree of conditioned response to the interpersonal conflict stimulus correlated with a higher ZAN-BPD total score. Fear conditioning and extinction can be successfully achieved, using interpersonal conflicts as a stimulus. Given that conditioned fear caused by the interpersonal conflicts is likely associated with borderline personality traits, this paradigm could contribute to further understanding of underlying mechanisms of interpersonal fear implicated in borderline personality disorder.

## Introduction

Anxiety and fear are indispensable emotional states for survival that serve as an adaptive function in a changing environment. These conditions are caused by a variety of stimuli, including not only physical stimuli but also social contextual stimuli such as interpersonal conflicts [1]. However, maladaptive anxiety and fear states can result in psychiatric illnesses such as anxiety disorders [2, 3]. In particular, social fear caused by interpersonal conflicts is associated with psychiatric illnesses such as some personality disorders, social anxiety disorder (SAD), and mood disorders [4]. For example, patients with SAD who suffer from fear of social interaction avoid social situations, resulting in remarkable distress in their life.

We have previously shown that abnormal neural plasticity underlies psychiatric disorders [5–7]. Aberrant learning (conditioning) processes of fear memory, one form of neural plasticity, are considered to play a pivotal role in the pathophysiology of anxiety disorders [8–10]. In order to understand neurobiological mechanisms underlying those disorders, fear-conditioning model has been widely used; this model is characterized by a form of associative learning in which contingencies are established by pairing aversive stimuli (an unconditioned stimulus [US]) with a previously neutral stimulus (a conditioned stimulus [CS]) [11]. Subtypes of anxiety disorders are primarily differentiated by the nature of CSs and USs in the fear conditioning model. In this context, distressing events, panic attacks, and traumatic encounters are regarded as USs that provoke unconditioned anxiety in patients with SAD, panic disorder, and posttraumatic stress disorder (PTSD), respectively, while corresponding CSs are people, places, and contexts [10]. Thus, it has been hypothesized that those who experience psychiatric symptoms would exhibit excessive conditioning and could not successfully achieve extinction of the learned association between CSs and USs.

In human fear conditioning studies, simple aversive stimuli such as an electrical shock and noise have been used as a US; in fact, these physical stimuli have been empirically applied to investigate the pathophysiology of anxiety disorders, borderline personality disorder (BPD), and PTSD [12–17]. However, patients with those conditions are affected by stresses induced by socially semantic stimuli including interpersonal relationships, rather than simple physical stimuli [18–20]. In rodents, the mechanisms underlying fear conditioning is well established [21–25]. However, it is extremely difficult to study fear conditioning with social context in experimental animals. Thus, investigations focusing on interpersonal stresses in human are clearly warranted to elucidate the pathophysiology of the psychiatric disorders. For example, patients with BPD experience intense abandonment fears, and their frantic efforts to avoid abandonment provoke inappropriate anger and impulsive behavior [26, 27]. They also show a pervasive pattern of instability and difficulty in changing the maladaptive behavior in interpersonal relationships [28–30]. BPD has been investigated using behavioral tasks, cognitive tasks, and functional neuroimaging in order to elucidate abnormalities in the processing of emotionally relevant stimuli [31–40]. Kamphausen et al. conducted an instructed fear task in female BPD patients and control subjects. They found that BPD patients compared to control subjects did not show any functional magnetic resonance imaging (fMRI) signal decrease of amygdala activity or relative ventromedial prefrontal cortex (vmPFC) activity increase. On the other hand, BPD patients showed increased connectivity of the amygdala with vmPFC but decreased connectivity of subgenual anterior cingulate cortex (ACC) with dorsal ACC compared to control subjects [41]. These studies have shown that BPD is characterized by heightened sensitivity and/or reactivity to emotional stimuli. Thus, an electrical shock or noise as a US is too simplistic to apply to the investigation of core mechanisms underlying these disorders. On the other hand, findings from research employing psychophysiological measures that can directly evaluate the subjective aspect of fear, conflict, and distress through autonomic response have been inconsistent [42–46]. They mainly used visual aversive stimuli, including disgusting pictures and unpleasant words to focus on simple emotional processing and dysregulation, which in turn made their results depend on subjects’ attention, commitment, and the self-relevance of negative stimuli [45]. Moreover, in these previous studies, they have not employed the fear conditioning paradigm other than two studies by Baskin-Sommers and Ebner-Priemer et al. [16, 17] despite the importance of the learning pattern in the psychopathology of BPD. Given that higher-order and complex social fear may play pivotal roles in the symptomatology of the aforementioned disorders like BPD and PTSD, these limitations clearly indicate the need of investigating interpersonal conflicts in the classic fear conditioning paradigm in order to elucidate the mechanisms. In addition, Davis et al. conducted fMRI imaging during a social conditioning task using face presentation paired with sentence presentation which has positive, neutral or negative social value [47]. However, sentence presentation without any prosody may not be sufficient to reflect interpersonal conflicts in the real world communication.

Therefore, we designed a conditioning experiment that used fear of interpersonal conflicts for the first time in order to apply it to future investigations of learning patterns in patients with anxiety disorders and BPD. In this study, we aimed to explore the feasibility of using the interpersonal stimuli as social stresses in the fear conditioning paradigm by investigating the achievement of associative learning in healthy individuals. In the stimuli, pictures of an actors’ face are presented with unpleasant verbal messages in order to cause interpersonal conflicts. We also employed two other USs that were conventionally used in previous studies on fear conditioning as control stimuli: an aversive sound to induce fear by direct physical stimuli (i.e. a US for classical fear conditioning) [48], and disgusting pictures from the International Affective Picture System (IAPS) [49] to induce semantic fear by non-social visual aversive stimuli [50]. Therefore, we employed three types of stimuli in this conditioning experiment in order to evoke different-order fear, which in turn make it possible to detect vulnerabilities specific to different disorders. We measured and compared the amplitudes of the skin conductance response (SCR) in the fear conditioning paradigm using these three types of USs involved in different-order fear. Changes in the SCR amplitudes were able to represent the magnitude of conditioned responses by three types of US. Further, we examined relationships between personality characteristics, especially the BPD trait, and the amplitudes of fear conditioning in response to the new stimuli.

## Materials and Methods

### Ethics Statement

**Fig 1:**
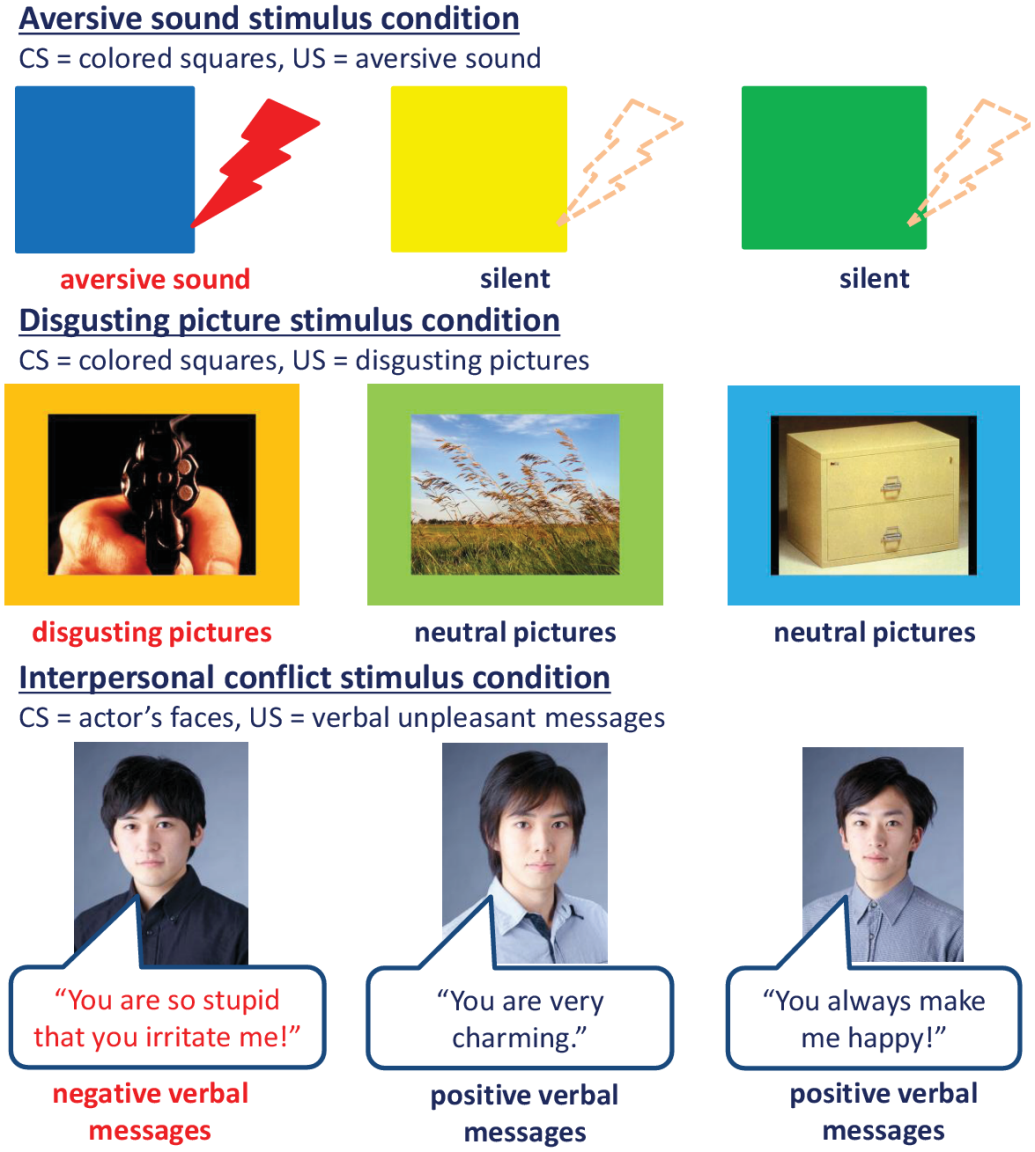
Conditioned stimulus and unconditioned stimulus used in the three types of stimulus paradigms. Abbreviations: CS, conditioned stimulus; US, unconditioned stimulus.

This study was conducted at Keio University School of Medicine, Tokyo, Japan. It was approved by the institutional review board of Keio University School of Medicine, and participants provided a written informed consent prior to their study entry. This study was registered at the UMIN Clinical Trials Registry as UMIN000004900. The individuals shown in Fig 1 have given written informed consent (as outlined in PLOS consent form) to publish their images.

### Participants

Twenty-nine healthy females were recruited from a local community. Inclusion criteria of participants were females aged 18 years or older who were capable of providing informed consent for participation in this study. Exclusion criteria were a) presence of any Axis I or II mental disorders based on the Diagnostic and Statistical Manual of Mental Disorders 4th Edition (DSM-IV)[51], b) presence of currently unstable physical illnesses, including neurological disorders, or c) current use of a beta-blocker or beta-stimulator.

Participants randomly underwent fear conditioning and extinction procedures in response to the following three types of stimuli, respectively: (1) a simple aversive sound, (2) disgusting pictures, and (3) pictures of an actors’ face with a negative verbal message that was designed to cause interpersonal conflicts. Changes in the SCR amplitudes in response to those stimuli were measured as an autonomic outcome of conditioned responses.

### Apparatus

The visual stimuli were presented over a black background on a 23-inch computer monitor. Participants were seated at a distance of 60cm from the computer monitor. The auditory stimuli were delivered through headphones with a noise-canceling effect. The stimuli presentation was implemented using the SuperLab software.

### Experimental procedures

Participants underwent behavioral experiment procedures in three types of stimulus conditions. They were given a 10-minute break between these procedures. The experimental procedures were designed based on the procedure by Bechara et al [52, 53]. Each procedure consisted of three consecutive phases: habituation, acquisition, and extinction. In each procedure, participants were presented three CSs and an aversive US for conditioning during the acquisition phase (Fig 1). The habituation phase comprised six trials in which the three CSs were presented in a randomized order (two trials of each CS). In the acquisition phase, one CS was randomly selected from these three CSs and the selected CS was paired with the US at a partial reinforcement rate of 50% (i.e. CS+). The other two CSs were presented without paring the US during the acquisition phase (i.e. CS-). The acquisition phase included 36 trials in which a CS+ and CS-s (12 trials of each CS) were presented in pseudo-randomized order. The pseudo-randomized order was designed to demonstrate the CS+ paired with the US as the first stimulus and the CS+ without pairing the US as the last stimulus when the CS+ was shown during the acquisition phase. The US occurred 3.0 seconds after the beginning of the presentation of CS+s in the CS+ trials. The disappearance of the visual stimulus of CSs was set in synchronization with the end of the US. In the extinction phase, all the CSs were presented without pairing the US. The extinction phase consisted of 18 trials (6 trials of each CS) in a randomized order. In all the experimental phases, an inter-trial interval (i.e. the duration from the disappearance of the visual stimulus to the appearance of the following one) was set at 10 seconds. Before the experiments, participants were instructed that they may or may not receive a US after the CS presentation. The order of each procedure was randomized in each participant.

### Stimuli

**Fig 2:**
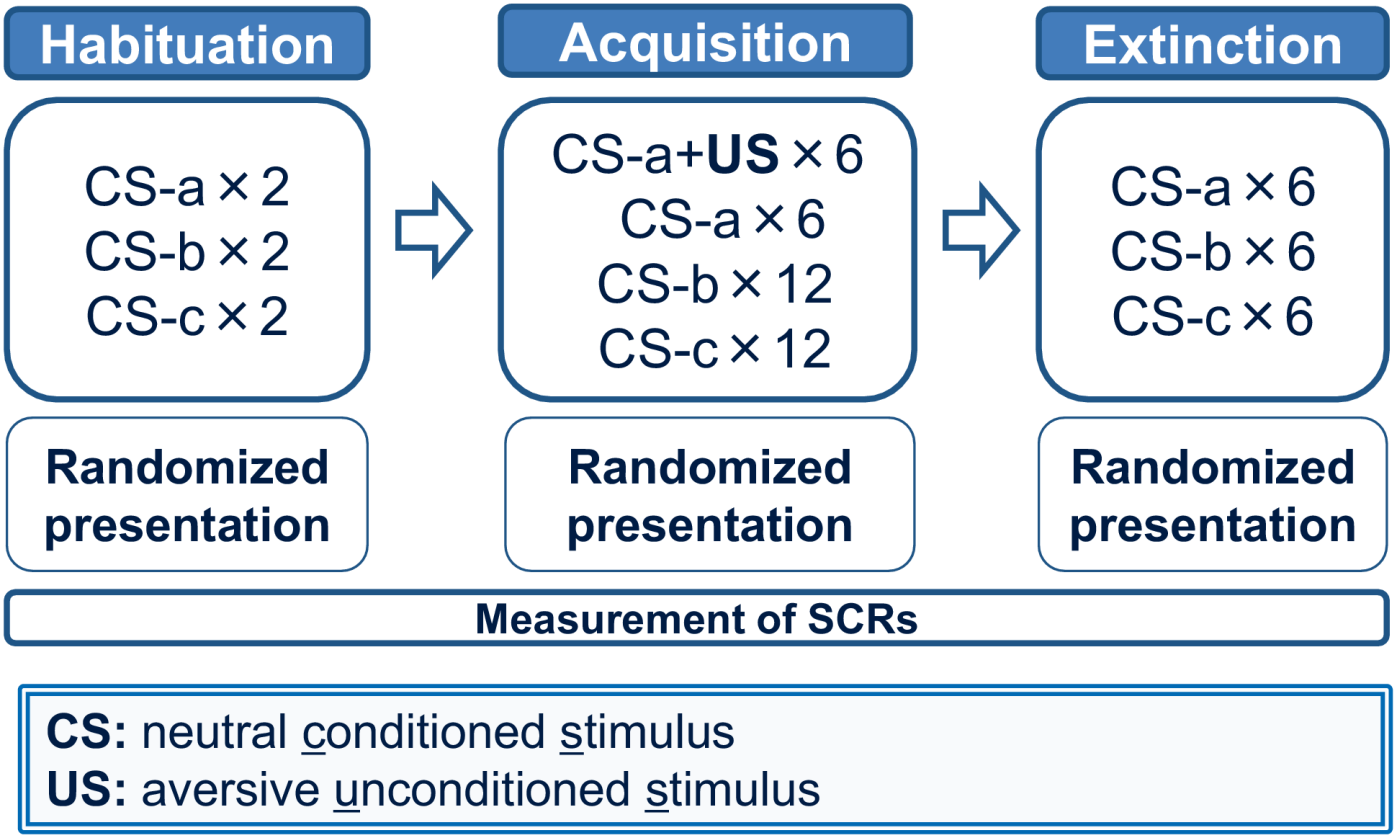
Fear conditioning procedure in the present study. The behavioral procedure consisted of three consecutive phases: habituation, acquisition, and extinction. Subjects received three CSs and an aversive US. The habituation phase comprised 6 trials (2 trials of each CS). In the acquisition phase, one CS was randomly selected from those three CSs and the selected CS was then paired with the US at a partial reinforcement rate of 50% (i.e. CS+). Other two CSs were presented without pairing the US during the acquisition phase (i.e. CS-). The acquisition phase included 36 trials (12 trials of each CS). The extinction phase consisted of 18 trials (6 trials of each CS).

In the aversive sound stimulus condition, three different colored squares were used as CSs and an aversive sound (95 dB, 2.5 seconds) was used as a US. In the disgusting picture stimulus condition, three different colored squares, whose colors were different from the three colors employed in the aversive sound stimulus condition, were used as CSs and paired with neutral pictures from the IAPS when CSs were not paired with US. Unpleasant pictures from the IAPS were used as USs. Those pictures were selected based on mean arousal dimension scores in the IAPS; neutral pictures had an arousal score ranging within the mean±1 standard deviation (*SD*), and unpleasant pictures had an arousal score of higher than mean+2*SD*. Because of ethical considerations, we excluded extremely invasive pictures such as *mutilation*, *baby tumor*, and *hanging* from the unpleasant pictures as USs. Two colored squares were paired with neutral pictures as the CS-s while the remaining one colored square paired with unpleasant or neutral pictures was used as the CS+. These colors were selected to have similar values in luminosity and saturation. In the interpersonal stimulus condition, pictures of three actors’ faces were used as CSs and paired with positive verbal messages when CSs were not paired with US. Negative verbal messages were used as USs (Fig 2). Beforehand, twenty female volunteers rated their impression on the pleasantness of six actors’ pictures and the 120 verbal messages, using a scale of from 1 (the most unpleasant) to 9 (the most pleasant). Three actors’ pictures having average pleasantness (i.e. within the range of the mean ± 1*SD* in the scale) were chosen from the six actors’ pictures as CSs. The six most negative verbal messages were chosen from the 20 unpleasant messages based on the scale score. The sixty most positive messages were also selected from the 100 pleasant messages based on the scale score, and divided into 3 groups to have equivalently averaged scores. The verbal messages were beforehand recorded and designed to last 1.5–2.5 seconds at 50–60 dB.

### Physiological recordings

Electrodermal activity was evaluated by means of skin conductance recording. Throughout the experiments, the amplitude of the SCR was recorded using two electrodes (8mm; Ag/AgCl) with a recording device (Polymate, TEAC, Tokyo, Japan). The electrodes were attached to the forefinger and middle finger of the left hand of participants. Before electrode attachment, skin was carefully cleaned with alcohol. Skin conductance signals were digitized and analyzed by means of the Ledalab 3.4 software (http://www.ledalab.de/) to extract a phasic electrodermal activity based on continuous decomposition analysis [54]. The criterion for the smallest scorable SCR was set at 0.01 μS. SCR was measured within a window of 1–8 second after visual stimulus onset. We used the averaged event-related SCR amplitudes of all of the CS+ trials not paired with the US during the acquisition phase to evaluate the conditioning. The averaged event-related SCR amplitudes in the first and last three CS+ trials during the extinction phase were also used to evaluate the extinction of conditioned fear.

### Clinical Assessment

We conducted a self-administered questionnaire about the history of sexual abuse or other trauma of subjects. Participants were also asked to evaluate the faces of those three actors on a 9-point Likert scale (1 = the most unpleasant to 9 = the most pleasant) before they underwent experimental procedures. Personality traits and clinical characteristics of the participants were assessed with the Emotion Regulation Questionnaire (ERQ) [55], the Revised NEO Personality Inventory (NEO-PI-R) [56], the Symptom Checklist 90-R (SCL-90-R) [57], and the Zanarini Rating Scale for Borderline Personality Disorder (ZAN-BPD) [58]. The ERQ is a questionnaire that includes 10 questions with a score of 1 to 7 for each question to assess individual differences in the habitual use of two emotion regulation strategies: cognitive reappraisal and expressive suppression. Cognitive reappraisal score ranges between 6 and 42, and expressive suppression score ranges between 4 and 28; a greater value indicates a greater trait of emotion regulation strategy. The NEO-PI-R is a psychological personality inventory that includes 240 questions rated according to the five grade evaluation system from “strongly agree” to “strongly disagree” for the five personality traits: neuroticism, extraversion, openness, agreeableness, and conscientiousness to experience. Each score ranges between 19 and 80; a greater value indicates a greater trait. The SCL-90-R is an instrument that is used to evaluate a broad range of psychological problems and symptoms of psychopathology. A total score ranges between 0 and 360, and a greater value indicates more psychological problems. The ZAN-BPD is a nine-item, clinician-administered scale to assess the severity of DSM-IV-based BPD symptoms. A total score ranges between 0 and 36, and a greater score indicates more severe BPD symptomatology.

### Statistical Analysis

The changes in the SCR amplitudes were compared between CS+ and CS- during both the acquisition and extinction phases in each stimulus by paired t-tests to test a difference of the mean amplitudes of the SCR. One-way analysis of variance (ANOVA) was used for comparison of “the differential SCR” (i.e. a difference in the changes in the SCR amplitudes in response to CS+ subtracted by the changes in the SCR amplitudes in response to CS-) among those three stimuli to test difference of conditioned response [59, 60]. Moreover, multiple linear regression analysis with force entry was performed to examine the differential SCR and total score in the ERQ, NEO-PI-R, SCL-90-R, and ZAN-BPD. Significance level was two-sided 5% for all tests. Multiple comparisons were conducted with Bonferroni correction. All statistical analyses were conducted using IBM SPSS Statistics version 21 for Windows (IBM Corporation, New York). Values in results are presented as mean ± *SD*.

## Results

### Characteristics of the participants

Demographic and clinical characteristics of the twenty-nine participants are summarized in Table 1. No subject reported a history of severe trauma including sexual abuse. No significant difference was found among the scores rating those three actor’s faces.

**Table 1:** Demographic and clinical characteristics of subjects.

Characteristics	Subjects (*n* = 29)mean ± *SD* (range)
Age, years	24.0 ± 3.1 (20.0–32.0)
ERQ scores	
Cognitive reappraisal	29.0 ± 5.1 (21–39)
Expressive suppression	14.0 ± 4.7 (6–28)
NEO-PI-R scores	
Neuroticism	57.0 ± 13.0 (33–80)
Extraversion	50.0 ± 10.0 (27–69)
Openness	57.0 ± 9.1 (35–67)
Agreeableness	43.0 ± 13.0 (19–69)
Conscientiousness	44.0 ± 12.0 (19–66)
SCL-90-R total score	39.0 ± 39.0 (1–148)
ZAN-BPD total score	2.8 ± 4.7 (0–22)

Abbreviations: SD, standard deviation; ERQ, Emotion Regulation Questionnaire; NEO-PI-R, Revised NEO Personality Inventory; SCL-90-R, Symptom Checklist 90-R; ZAN-BPD, Zanarini Rating Scale for Borderline Personality Disorder.

### Conditioning and extinction

**Fig 3:**
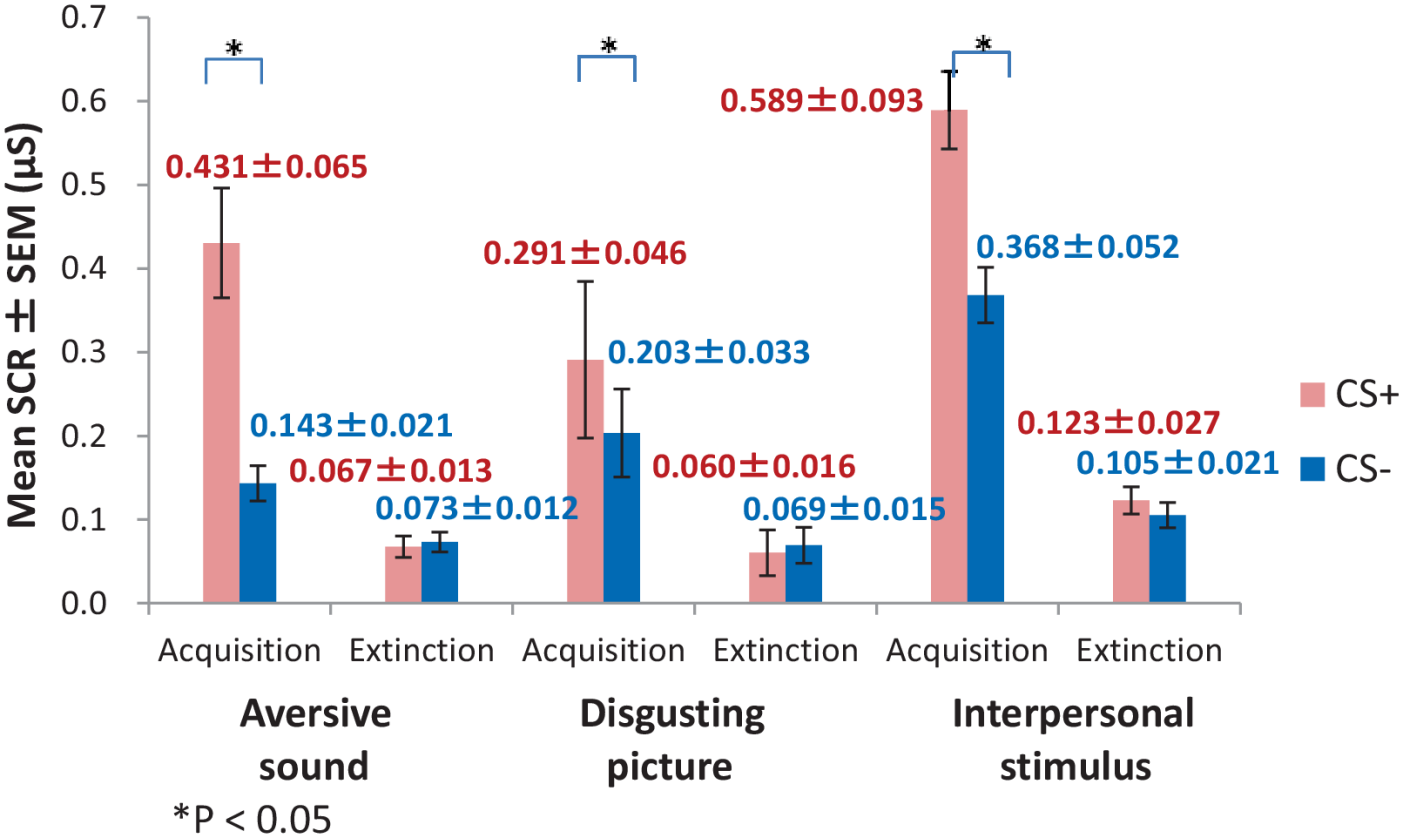
Mean SCR amplitudes during the acquisition and extinction phases in the three conditions. Abbreviations: SCR, skin conductance response; SEM, standard error of the mean; μS, microsiemens

Participants successfully showed a fear conditioning in all three of the conditions. The mean changes in the SCR amplitudes in response to CS+ were greater than those in response to CS- during the acquisition phase (CS+ = 0.43, CS- = 0.14, by the aversive sound, [*t*_(28)_ = 5.98, *p* < .001]; CS+ = 0.29, CS- = 0.20, by the disgusting pictures, [*t*_(28)_ = 2.28, *p* = .03]; and CS+ = 0.59, CS- = 0.37, by the interpersonal stimulus, [*t*_(28)_ = 3.33, *p* = 0.002], respectively) (Fig 3). Subsequently, participants successfully extinguished conditioned fear responses in all three of the conditions (CS+ = 0.07, CS- = 0.07, by the aversive sound, [*t*_(28)_ = -.50, *p* = .62]; CS+ = 0.06, CS- = 0.07, by the disgusting pictures, [*t*_(28)_ = -.72, *p* = .48]; and CS+ = 0.12, CS- = 0.11, by the interpersonal stimulus, [*t*_(28)_ = 1.17, *p* = .25], respectively) (Fig 3).

#### Comparison of differential SCRs among the three stimulus conditions

A one-way ANOVA showed that the differential SCR during the acquisition phase significantly differed among the three stimulus conditions (*F*_(2, 84)_ = 4.00, *p* = .02); however, Bonferroni post-hoc comparisons of the three conditions found no significant difference between the interpersonal stimulus condition (mean ± *SD* = 0.22 ± 0.33 μS) and either of the other two conditions (vs. the aversive sound stimulus (0.29 ± 0.25 μS), *p* = 1.00; vs. the disgusting picture stimulus (0.09 ± 0.20 μS), *p* = .20). During the extinction phase, no significant differences were found in the differential SCR among the three stimulus conditions (*F*_(2, 84)_ = 1.22, *p* = .30).

#### Relationship between personality traits of participants and conditioning

In the interpersonal stimulus condition, the ZAN-BPD total score was positively associated with the differential SCR during the acquisition phase (*β* = .82, *p* = .02) (Table 2). Correlational analysis also demonstrated an association between the ZAN-BPD total score and differential SCR during the acquisition phase (*R* ^*2*^ = .406, *p* = .029). However, the association failed to reach any statistical significance after Bonferroni correction. The ERQ, NEO-PI-R, and SCL-90R total score failed to show any association with the differential SCR during the acquisition phase in all three of the conditions.

**Table 2:** Results of multiple linear regression analysis with differential SCR during the acquisition phase as a dependent variable in the interpersonal stimulus condition.

	Differential SCR during the acquisition phase		
Factors	*β*	*P*-value	Unstandardized coefficient 95% *CI*
ERQ scores (unit = 1)			
Cognitive reappraisal	< .01	.99	-.032 - .032
Expressive suppression	.24	.33	-.019 - .052
NEO-PI-R scores (unit = 1)			
Neuroticism	.06	.85	-.015 - .018
Extraversion	.23	.48	-.014 - .029
Openness	.12	.56	-.012 - .021
Agreeableness	.31	.21	-.005 - .021
Conscientiousness	-.11	.78	-.026 - .020
SCL-90-R total score (unit = 1)	-.81	.07	-.014 - .001
ZAN-BPD total score (unit = 1)	.82	.02*	.010 - .102

* *p* < .05

Abbreviations: ERQ, Emotion Regulation Questionnaire; NEO-PI-R, Revised NEO Personality Inventory; SCL-90-R, Symptom Checklist 90-R; ZAN-BPD, Zanarini Rating Scale for Borderline Personality Disorder.

#### Relationship between personality traits of participants and early and late extinction

During the first three extinction trials in the interpersonal stimulus condition as an index of the onset of extinction, the ZAN-BPD total score was positively associated with the differential SCR (*β* = .82, *p* < .001) (Table 3). Correlational analysis also showed a significant association between the ZAN-BPD total score and differential SCR during the early extinction phase (*R* ^*2*^ = .755, *p* < .001) after Bonferroni correction. In contrast, none of the ERQ, NEO-PI-R, SCL-90R, and ZAN-BPD scores was associated with the differential SCR during the first three extinction trials in the aversive sound or the disgusting picture stimulus conditions. On the other hand, none of the ERQ, NEO-PI-R, SCL-90R, and ZAN-BPD scores was associated with the differential SCR during the last three extinction trials, as an index of the successful extinction, in all three of the conditions.

**Table 3:** Results of multiple linear regression analysis with differential SCR during the early extinction phase as a dependent variable in the interpersonal stimulus condition.

	Differential SCR during the early extinction phase		
Factors	*β*	*P*-value	Unstandardized coefficient 95% *CI*
ERQ scores (unit = 1)			
Cognitive reappraisal	-.24	.11	-.019 - .002
Expressive suppression	.27	.08	-.002 - .022
NEO-PI-R scores (unit = 1)			
Neuroticism	-.04	.85	-.006 - .005
Extraversion	.35	.09	-.001 - .013
Openness	.09	.48	-.003 - .007
Agreeableness	-.07	.60	-.005 - .003
Conscientiousness	-.28	.24	-.012 - .003
SCL-90-R total score (unit = 1)	.05	.85	-.002 - .003
ZAN-BPD total score (unit = 1)	.82	< .001*	.016 - .045

* *p* < .05

Abbreviations: ERQ, Emotion Regulation Questionnaire; NEO-PI-R, Revised NEO Personality Inventory; SCL-90-R, Symptom Checklist 90-R; ZAN-BPD, Zanarini Rating Scale for Borderline Personality Disorder.

## Discussion

This is the first study to demonstrate that interpersonal conflicts successfully exhibited fear conditioning in healthy individuals, which was subsequently extinguished in our fear conditioning experiment in a similar manner to the conventional stimuli such as a simple aversive sound and disgusting pictures. Moreover, those who had a greater tendency of borderline personality trait were associated with a higher conditioned fear response and a greater difficulty of extinction during the early extinction phase when the interpersonal conflict stimulus was employed. In contrast, such relationships were not observed in the other conditions. This is the first study to employ realistic interpersonal communication in the fear conditioning paradigm and evoke the emotional reactivity by the interpersonal conflict stimulus in healthy individuals. Furthermore, our paradigm adopted the classical fear conditioning and successfully demonstrated the associative learning in the interpersonal communication setting as is the case with simple physical stimuli. Thus, these results have confirmed the feasibility of our fear conditioning paradigm employing interpersonal conflicts as an aversive stimulus, which can be applied to future studies for psychiatric disorders implicated in interpersonal fear such as BPD and anxiety disorders. Further research is expected to investigate psychiatric conditions involved with interpersonal fear, using our newly developed experiment.

As expected, this study successfully replicated the fear conditioning induced by both the aversive sound and disgusting picture stimulus [50, 52]. Only the social interpersonal fear, however, demonstrated the relationship between the borderline personality trait, and conditioning and extinction. A simple aversive sound and disgusting pictures have been thought to induce fear by direct physical stimuli and visual semantic stress from the environment in the context of visual information, respectively. On the other hand, the interpersonal conflict stimulus was designed to evoke comprehensive social fear induced by interpersonal conflicts in our experiment. In light of the different nature of such a potentially higher level of fear caused by interpersonal conflicts, it is reasonable to observe the fact those relationships between the borderline personality trait and degrees of conditioning and extinction were only detected by the interpersonal stimulus condition. Consequently, the results in the present study clearly support the unique feasibility of our fear conditioning task to elucidate interpersonal fear implicated in BPD.

To our knowledge, few psychophysiological studies used the fear conditioning paradigm to evaluate patients with BPD [16]. It is advantageous to use the fear conditioning paradigm in human experiments in that they explicitly exhibit autonomic arousal and link findings with animal studies in the identical paradigms. Baskin-Sommers et al. employed fear-potentiated startle (FPS) as a psychophysiological measure and an electrical shock as a US. They found that women with a higher score on the personality assessment inventory-borderline features scale showed a greater degree of FPS when they were required to focus their attention and commitment on the threat-relevant dimension of the experimental stimuli [16]. The authors concluded that attention should be taken into account to elucidate the inconsistent evidence on emotional reactivity in psychophysiological studies on BPD. In our study, we uniquely employed the interpersonal conflict stimulus, which often triggers unstable and intense interpersonal relationships in patients with BPD, in the fear conditioning paradigm. The interpersonal conflict stimulus could draw more attention and evoke more emotional reactivity in social and interpersonal contexts than simple visual aversive stimuli. As a result, we successfully demonstrated the relationships between borderline personality trait, and fear conditioning and extinction in healthy participants. Therefore, it is expected that our experiment has the potential to evaluate abnormal learning processes of interpersonal conflicts in patients with BPD. In addition, it will be highly relevant to investigate extinction recall and renewal to estimate the long-term effects of the learning process in future investigations.

This study should be interpreted with several limitations. First, study participants are limited to female Mongoloid. The generalizability of the findings to different populations such as males and people from different ethnic backgrounds may be limited. We enrolled only female participants because this study was designed to demonstrate the feasibility of a new interpersonal fear conditioning paradigm for future research on BPD which is diagnosed predominantly in females. In addition, autonomic response was reported to have a sex difference in previous studies [61, 62]. Second, static images accompanied by spoken messages with prosody used in the present study may be less potent to activate emotional processing than video interpersonal stimuli. We decided to use static images instead of video interpersonal stimuli because use of static images was considered an established methodology in fear conditioning paradigm whereas it was not the case for video stimuli. Use of video stimuli, which was more complex, would have involved difficulties in controlling experimental conditions. However, such video interpersonal stimuli may be more potent and should be considered in future studies. Third, positive messages were paired with CS- in the interpersonal stimulus although it may be argued that neutral messages should be used instead. People with BPD find it difficult to erase their unpleasant inter-personal memory, which is more prominent when it is caused by people that they used to have a good relationship with than those who are emotionally neutral to them. Therefore, we think that the contrast between positive and negative values of messages more likely reflect interpersonal conflicts in the real world communication that people with BPD experience, compared to that between neutral and negative messages. On the other hand, positive messages may be perceived as threatening in patients with BPD because these positive messages may be incongruent to their self-image or may signal potential loss, which should be taken into account when the data are interpreted. Forth, we did not conduct any procedure to investigate brain function such as fMRI or event-related potential (ERP) in this study. Therefore, we were unable to investigate the mechanisms underlying the results that we observed. Further investigations on social emotional brain processing and hemispherical brain processing are clearly warranted to elucidate the specific emotional processing and learning mechanism of patients with BPD [63, 64].

## Conclusion

Our original interpersonal experiment can be successfully applied to studies employing the fear conditioning paradigm. In addition, those who have a greater degree of borderline personality trait show a stronger conditioned autonomic response to the interpersonal stimulus whereas the other stimuli was not able to detect such a relationship. Thus, this experiment has the potential to detect the abnormality of fear conditioning induced by higher-order interpersonal conflicts, which is assumed to involve higher neural systems than those in response to simple auditory and visual stimuli, in people who suffer from psychiatric conditions implicated in interpersonal fear, including BPD.
